# ﻿Water beetles of northeastern Algeria: new records for the country and faunistic updates (Coleoptera, aquatic Adephaga, Dryopidae, Hydrophiloidea, Hydraenidae)

**DOI:** 10.3897/zookeys.1248.153053

**Published:** 2025-08-07

**Authors:** Khaoula Mahmoudi, Mario E. Toledo, Fatiha Bendali-Saoudi, Ilaria Negri

**Affiliations:** 1 Department of Natural and Life Sciences, Faculty of Sciences, Echahid Cheikh Larbi University, Tebessa, Algeria Echahid Cheikh Larbi University Tebessa Algeria; 2 Department of Sustainable Crop Production (DI.PRO.VE.S.), Università Cattolica del Sacro Cuore, Piacenza, Italy Università Cattolica del Sacro Cuore Piacenza Italy; 3 Laboratory of Applied Animal Biology, Department of Biology, Faculty of Sciences, Badji Mokhtar University, Annaba, Algeria Badji Mokhtar University Annaba Algeria

**Keywords:** Algeria, checklist, faunistic, new records, taxonomy, water beetles

## Abstract

Water beetles collected from Lake Tonga (North-East of Algeria), one of the best preserved and biodiverse coastal habitats in North Africa, have been studied and identifications of species reassessed, since most previous determinations published in Mahmoudi et al. (2023) were found to be incorrect. In this paper a revised species list is provided, with a small batch of previously unidentified material collected from a second biotope, Garaat Djamel, located 60 km west of Lake Tonga. A total of 42 species were identified, belonging to the families Gyrinidae, Haliplidae, Noteridae, Hygrobiidae, Dytiscidae, Dryopidae, Helophoridae, Hydrochidae, Hydrophilidae, and Hydraenidae, hosting approximately 13% of the Algerian aquatic beetle fauna, with the vast majority of this diversity concentrated in Lake Tonga, underscoring its status as a key hotspot of aquatic beetle biodiversity in the region. Five of the identified species are new records for Algeria: Helophoruscf.paraminutus Angus, 1986, *Amphiopssenegalensis* (Laporte, 1840), *Enochrusnatalensis* (Gemminger & Harold, 1868), *Crephelochares ?livornicus* (Kuwert, 1890) and *Ochthebiusfallaciosus* Ganglbauer, 1901. *Hydrochusgrandicollis* Kiesenwetter, 1870, and *Coelostomahispanicum* (Küster, 1848), recently recorded from Algeria, but omitted from major catalogues, are here confirmed for the country. Furthermore, the discovery of a male of the poorly known *Haliplusruficeps* Chevrolat, 1806 represents the first documented record of this species in more than a century. Photographs of the habitus and male genitalia of *H.ruficeps* and of the newly recorded species are provided. At present, Algeria supports 301 species of aquatic Coleoptera across 84 genera and 17 families. A comprehensive and updated checklist is also given, with a discussion on several doubtful or unconfirmed records.

## ﻿Introduction

Despite a body of literature on the topic dating back to the second half of the 19^th^ century, and Algeria’s central geographical location in western North Africa, knowledge of water beetles in the country is currently rather limited (Lamine et al. 2019). This is not only in comparison with the European side of the Mediterranean basin, but also to neighbouring Morocco and Tunisia, which have seen an increase in publications in recent years, thanks in part to cooperation with European specialists (Angus and Aouad 2009; Touaylia et al. 2009, 2010a, b, 2011a, b, 2013; Taybi et al. 2017; Touaylia 2017; Mabrouki et al. 2018; Guellaf et al. 2021; Benamar et al. 2021a, b,c, 2022a, b; Belhaj et al. 2023, 2024). In the early 2000s, a Turkish-Algerian collaboration produced several articles focused on northern Algeria, including new records for the national territory (Bouzid and İncekara 2006; İncekara et al. 2007; İncekara and Bouzid 2007a, b; İncekara 2008) and more recently additional publications have provided new checklists for northern Algeria (Boukli Hacene et al. 2012; Lamine et al. 2019, 2022; Mahmoudi et al. 2023). Unfortunately, many of these works contain inaccuracies, errors of identification or at least identifications that require verification; this is probably due to limited cooperation with leading specialists, with only a few exceptions (e.g. Fery and Bouzid 2016).

To date, the most comprehensive references for Algerian aquatic Coleoptera are the three volumes of the revised and updated edition of the Catalogue of Palearctic Coleoptera (Löbl and Löbl 2015, 2016, 2017), along with subsequent updates for some taxonomic groups, available online or privately issued (Mascagni 2020; Hájek and Fery 2022; Przewózny 2022; Nilsson and Hájek 2024, 2025). It is interesting to note that some new records for the country published in the above-mentioned papers, even when credible, have not been included in latest updates of the Catalogue (Przewoźny 2022; Nilsson and Hájek 2025) (see in Suppl. materials 1, 2). Lamine et al. (2022) mention the difficulty of identifying North African aquatic beetles to species level, with the exception of Hydraenidae and Elmidae (see Lamine et al. 2019). This reveals the challenges faced by local researchers, including limited access to valid and updated instruments for the identification and systematics of most groups of aquatic beetles, insufficient collaboration with key foreign experts, and the general scarcity of research in the area (Lamine et al. 2019; Mahmoudi et al. 2023).

A recent eco-faunistic study by Mahmoudi et al. (2023) on the water beetles of Lake Tonga (Garaat Tonga) in north-eastern Algeria is particularly noteworthy. Based on the absence of prior literature, a survey on the aquatic Coleoptera of this biotope had never been carried out before. However, the checklist in this paper contains several misidentifications, listing some species whose presence in this geographical area is highly improbable or even impossible [*Haliplusflavicollis* Sturm, 1834, *Agabusbifarius* (Kirby, 1836), *Leiodyteshieroglyphicus* (Régimbart, 1893), *Helophorusflavipes* Fabricius, 1792, *Hydrochuselongatus* (Schaller, 1783), *Berosusinfuscatus* LeConte, 1855].

During a scientific cooperation between the University of Tebessa (Algeria) and the Università Cattolica del Sacro Cuore in Piacenza (Italy), we had the opportunity to re-examine part of the material on which that article was based, together with another small batch of unidentified water beetles from a different locality. This led us to the discovery of some interesting faunistic data, including records of new species for Algeria and the confirmation of others.

The terms “water beetles” or “aquatic Coleoptera” are herein used in sensu lato. However, the families considered in this study are strictly or predominantly aquatic and they are all included in the “True Water Beetles” sensu Jäch (1998) and Jäch and Balke (2008). While some species of Hydrophilidae (all belonging to the subfamily Sphaeridiinae) are secondarily terrestrial, these are not excluded from the checklist of Algerian aquatic Coleoptera (Suppl. material 1), as they belong to a family that is fundamentally and predominantly aquatic.

## ﻿Materials and methods

**Figure 1.**
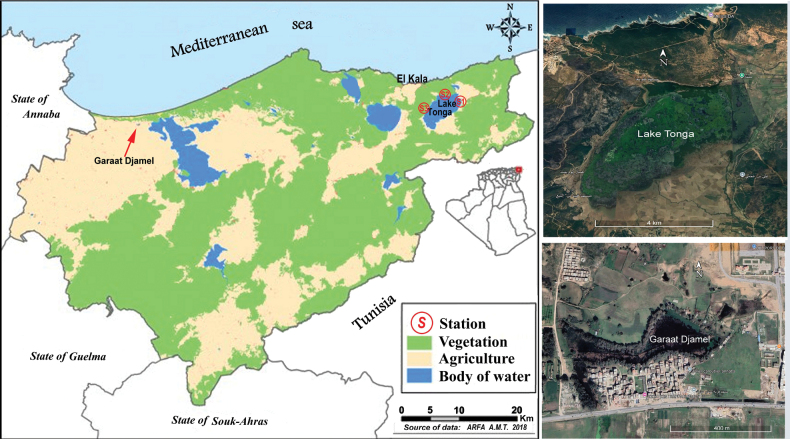
Geographical location of Garaat Djamel and Lake Tonga in El Taref State, northeast Algeria (from Mahmoudi et al. 2023, modified), and satellite photos of the two lakes. S1, S2, and S3 indicate the sampling stations at Lake Tonga in Mahmoudi et al. (2023).

The sampling periods, field collection methods, preservation of the specimens, as well as a description of the biotope of Lake Tonga, are detailed in Mahmoudi et al. (2023, 2024). The re-examined material from this locality, which forms the basis of the present study, consists of 592 specimens of water beetles stored in 23 vials filled with glycerol, almost all bearing an identification label at species level (noted in quotation marks in the text). However, as most of these identifications were incorrect, during the revision of this material the original labels were replaced with new ones with the corrected specific names. Additional material studied includes a second lot of unidentified water beetles, consisting of 43 specimens preserved in 70% ethanol, and collected from a small lake (Garaat Djamel) ca 60 km West of Lake Tonga, near the town of Annaba. The locations of both Lake Tonga and Garaat Djamel are illustrated in Fig. 1.

The study of this material was carried out under two different stereo microscopes: a Zeiss Discovery V8 equipped with two separated led spots and an Amscope SM-4T with ring led illumination. Some specimens were dissected, and the genitalia studied in wet condition (lactic acid, then passed in glycerol) with an Amscope SME-F8BH compound microscope. Photographs of both habitus and genital pieces were taken with an Amscope MU100 digital camera, mounted on both Amscope stereo and compound microscopes. All illustrations were retouched with Adobe Photoshop Elements 2021 software. The identification of species and the determination of their distributions refer to Guignot (1959a, b); Olmi (1972, 1976); Franciscolo (1979); Foster (1984); Angus (1986); Hansen (1987); Hansen and Hebauer (1988); Schödl (1991, 1993); Fery (1995); van Vondel and Dettner (1997); Hebauer (1998, 2002); Mascagni (2005); Rocchi (2005a, b); Jäch and Delgado (2008); Angus and Aouad (2009); Touaylia et al. (2009); Fery and Bouzid (2016); Kodada and Jäch (2016); Hájek (2017a, b); van Vondel (2017); Benamar et al. (2022a, b, 2024); Queney and Prévost (2021); Przewózny (2022); Toledo (2022); Shatrovskiy and Angus (2024); Nilsson and Hájek (2024, 2025). Chorotypes refer to Vigna Taglianti et al. (1999).

All the examined material is deposed in the collections of the Echahid Cheikh Larbi University of Tebessa, Algeria (**ECLUT**), except a few specimens retained for further study in the collections of the Università Cattolica del Sacro Cuore in Piacenza, Italy (**UCSCP**).

## ﻿Results

### ﻿Water beetles collected from Lake Tonga and Garaat Djamel

Compared to the 1,202 reported in Mahmoudi et al. (2023), the 592 specimens of water beetles studied from Lake Tonga, represent just more than half of the total material collected during the investigation of this biotope. In addition to being numerically different, these specimens were no longer sorted by sampling season, and were probably partly mixed after initial examination, since some vials, labelled with a single specific name, contained two or more species together. These factors make it difficult to directly compare the species determined here with those listed in Mahmoudi et al. (2023) and a meaningful comparison with the ecological data provided in that publication is impossible. With this premise we can provide only a checklist of the newly identified species, with systematic and faunistic notes on the most relevant data. Despite the reduced sample size, 30 species of water beetles were identified in this material, belonging to nine families: Gyrinidae (1 sp.), Haliplidae (4 spp.), Noteridae (2 spp.), Hygrobiidae (1 sp.), Dytiscidae (8 spp.), Helophoridae (1 sp.), Hydrochidae (1 sp.), Hydrophilidae (11 spp.), and Hydraenidae (1 sp.). This represents an increase compared to the 24 species and seven families reported in Mahmoudi et al. (2023). In addition, our list also includes two species of Dryopidae collected from this biotope, a family not covered in the previous study.

Garaat Djamel (36°49'N, 7°54'E) is a small coastal lake ca 60 km W of Lake Tonga, located in the same region (El Tarf), two kilometres East of Echatt City, ca 15 km east of Annaba. The lake, 1300 m from the seashore, at an altitude of 16 m a.s.l., is fed by groundwater and during the dry season, in summer, it shrinks in a small puddle (Houmani et al. 2023). A small batch of water beetles was collected during the spring 2023, using the Dipping methods, as already illustrated in Mahmoudi (2023, 2024), together with the use of non-killing bottle-traps trigged with bloody meat. The collected specimens were preserved in 70% ethanol. This material studied comprises 16 species belonging to five families (Haliplidae: 1 sp., Noteridae: 1 sp., Dytiscidae: 7 spp., Dryopidae: 1 sp., Hydrophilidae: 6 spp.). The finding of the prothorax of an *Hydrochara* would represent the first record for the genus in Algeria, even though the determination of the species is not possible due to the fragmentary nature of the material.

The taxa of particular interest (all from Lake Tonga) are discussed below. A complete checklist of the species of water beetles collected from both Lake Tonga and Garaat Djamel, with the distribution and chorology, is given in Table 1.

**Table 1.** List of the species of water beetles identified from Lake Tonga and Garaat Djamel, based on currently available material (compare the Lake Tonga list with Mahmoudi et al. 2023): (*n*) number of specimens, (*) sex not checked; (!) first record for Algeria. Data on distribution and ecology from Rocchi (2005a, b); Mascagni (2005, 2020); Jäch and Delgado (2008); Przewózny (2022); Toledo (2022); Nilsson and Hájek (2025). Chorological categories follow Vigna Taglianti et al. (1999); Touaylia et al. (2011b).

Family / species	*n* (per locality)		General distribution	Ecology
	L. Tonga	G. Djamel		
**Gyrinidae**				
Gyrinus (Gyrinus) urinator Illiger, 1807	1 ♂		Euro-Mediterranean	flowing and still waters (streams, rivers, lakes)
**Haliplidae**				
Haliplus (Liaphlus) guttatus Aubé, 1836	1 ♂		Mediterranean	still waters (ponds, grassy ditches, marshes)
Haliplus (Neohaliplus) lineatocollis (Marsham, 1802)	24		West Palearctic	flowing and slow-flowing waters, in a wide range of habitat
Haliplus (Neohaliplus) ruficeps Chevrolat, 1806	1 ♂		North African	unknown, likely as the preceding species
*Peltodytescaesus* (Duftschidt, 1805)	2	1(*)	Turano-Euro-Mediterranean	still waters (ponds, lakes, marshes)
**Hygrobiidae**				
*Hygrobiahermanni* (Fabricius, 1775)	2		Euro-Mediterranean	still waters (clay ponds)
**Noteridae**				
*Canthydrussiculus* (Ragusa, 1882)	57	1(*)	West Mediterranean	still waters (ponds, marshes, puddles)
*Noteruslaevis* Sturm, 1834	153		West Mediterranean	still waters (ponds, marshes, puddles)
**Dytiscidae**				
Agabus (Gaurodytes) nebulosus (Forster, 1771)		7	West Palearctic	still waters (ponds, marshes, puddles, ditches)
Agabus (Gaurodytes) bipustulatus (Linnaeus, 1767)		2	Palearctic	flowing and still waters, in a wide range of habitat
Agabus (Gaurodytes) conspersus (Marsham, 1802)		7	Palearctic	still waters also brackish (ponds, marshes, puddles, ditches)
*Colymbetesfuscus* (Linnaeus, 1758)		1 ♀	West Palearctic	still water (ponds, marshes, puddles, ditches)
Cybister (Cybister) lateralimarginalis lateralimarginalis (De Geer, 1774)		1 ♂	Turano-Euro-Mediterranean	still waters (ponds, lakes, marshes, bogs)
Cybister (Cybister) tripunctatus africanus Laporte, 1835		1 ♀	Afrotropico-Mediterranean	still waters (marshes, ponds)
Hydaticus (Prodatycus) leander (Rossi, 1790)	1 ♂		Afrotropico-Mediterranean	still waters (ponds, marshes, pools, ditches)
*Hyphydrusaubei* Ganglbauer, 1892	1 ♀	4	Euro-Mediterranean	still waters (ponds, marshes, puddles, ditches)
*Hydrovatuscuspidatus* (Kunze, 1818)	1 (*)		Turano-Euro-Mediterranean	still waters (ponds, lakes, marshes, bogs)
*Graptodytespietrii* Normand, 1933	1♀		North African	flowing and slow-flowing waters (streams, springs, ditches, pools)
Hygrotus (Hygrotus) inaequalis (Fabricius, 1777)	14		Palearctic	still waters (ponds, marshes, ditches, lakes)
Hygrotus (Hygrotus) guineensis (Aubé, 1836)	14		Afrotropico-Mediterranean	still waters (ponds, marshes, puddles, ditches)
*Laccophiluspoecilus* Klug, 1834	4		Centralasiatic-Euro-Mediterranean	still waters (ponds, bogs, marshes)
*Laccophilusminutus* (Linnaeus, 1758)	39		Palearctico-Oriental	still waters, in a wide range of habitats
**Dryopidae**				
*Dryopsalgiricus* (Lucas, 1849)		1 ♂	Mediterranean	flowing and still waters
*Dryops* sp.	1 ♀			
*Dryopspeyerimhoffi* Bollow, 1939	1 ♂		North African	still waters, swamps
**Helophoridae**				
Helophorus (Rhopalohelophorus) cf. paraminutus Angus, 1986 (!)	1 ♂		Not available (see main text)	still waters
**Hydrochidae**				
*Hydrochusgrandicollis* Kiesenwetter, 1870	1 ♂, 1 ♀		Mediterraneo-Macaronesian	still and flowing waters (stream banks, rivers, puddles)
**Hydrophilidae**				
*Amphiopssenegalensis* (Laporte, 1840) (!)	12		Afrotropico-North African	still waters (ponds, marshes)
Berosus (Berosus) signaticollis (Charpentier, 1825)	13		West Palearctic	still waters (ponds, puddles, marshes)
Berosus (Berosus) affinis Brullé, 1835	172	3	Mediterranean	still or slow-flowing waters (ponds, puddles, ditches)
Berosus (Enoplurus) bispina Reiche & Saulcy, 1856		3	Mediterranean	still or slow-flowing waters (ponds, puddles, ditches)
*Limnoxenusniger* (Gmelin, 1790)	13		West Palearctic	still waters (ponds, marshes)
*Hydrobiusfuscipes* (Linnaeus, 1758)	1		Holarctic	still waters (ponds, swamps, marshes)
*Hydrochara* sp.		1 remain	Not available	still waters
Enochrus (Methydrus) natalensis (Gemminger & Harold, 1868) (!)	36		Subcosmopolitan	still waters (ponds, puddles, marshes)
Enochrus (Lumetus) politus (Küster, 1849)	1 ♀	1 ♀	Mediterranean	still waters, also brackish
*Helochareslividus* (Forster, 1771)	17	5	Euro-Mediterranean	still waters (ponds, swamps, marshes, ditches)
*Crephelochares ?livornicus* (Kuwert, 1890) (!)	2 ♀		Not available	still waters (ponds, marshes)
*Anacaenalutescens* (Stephens, 1829)	1 (*)		Holarctic	flowing and still waters (ponds, swamps, ditches, rivers)
*Coelostomahispanicum* (Küster, 1848)	1 (*)		Mediterraneo-Macaronesian	flowing waters (stream banks), rarely swamps
**Hydraenidae**				
Ochthebius (Ochthebius) fallaciosus Ganglbauer, 1901 (!)	2 ♂ 1 ♀		Euro-Mediterranean	still waters, also brackish

### ﻿Haliplus (Neohaliplus) ruficeps Chevrolat, 1806 (Haliplidae)

**Figures 2–9.**
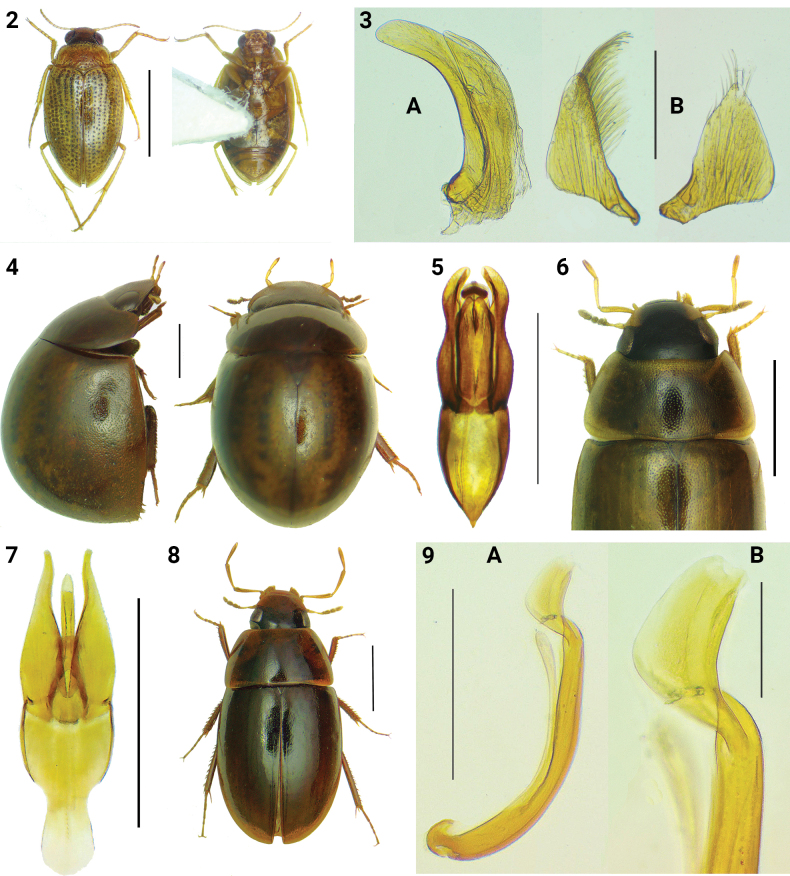
Water beetles of particular interest from Lake Tonga. **2***Haliplusruficeps*, habitus (dorsal and ventral aspect); **3***Haliplusruficeps*, aedeagus: **A.** Median lobe; **B.** Right and left parameres; **4.***Amphiopssenegalensis*, habitus (lateral and dorsal aspect); **5.***Amphiopssenegalensis*, aedeagus; **6.***Enochrusnatalensis* dorsal aspect (head, pronotum and basal half of elytra); **7.***Enochrusnatalensis*, aedeagus; **8.**Crephelochares?livornicus, female habitus; **9.***Ochthebiusfallaciosus*, aedeagus: **A.** Whole piece; **B.** Magnification of distal lobe. Scale bars: 1.00 mm (**2, 4–6, 8**); 0.50 mm (**7**); 0.20 mm (**3, 9A**); 0.05 mm (**9B**).

Among the numerous specimens of *H.lineatocollis* (Marsham, 1802) from Lake Tonga labelled “*Haliplusflavicollis* Sturm, 1834”, a single male differs distinctly in size (2.0 mm, compared to 2.4 mm of the smallest specimen of *H.lineatocollis* measured in this batch), as well as in morphological characters and colouration patterns. Externally, it matches the diagnosis given in Guignot (1959a) and van Vondel and Dettner (1997) for *H.ruficeps* (Fig. 2), despite the less blunt median lobe and the less elongated parameres (Fig. 3), compared to those illustrated in the latter publication. However, the left paramere is almost devoid of the club-shaped setae typically present in *H.lineatocollis*. *Haliplusruficeps* is one of the rarest and least-known taxa of Palearctic Haliplidae, for which no new data on distribution have been published since the first decades of the last century (Nilsson and van Vondel 2005). While *Haliplusruficeps* is currently recognized as a valid species, distinct from the closely related *H.lineatocollis*, information on its systematic status remains limited and no data are available on its variability.

**Distribution.** The range of this taxon is also rather unclear. It is currently considered an exclusively North African species (Algeria, Morocco: van Vondel 2017) since all previous records from Mediterranean Europe have been questioned by van Vondel and Dettner (1997). However, the systematic and the geonomy of this beetle require further investigation based on additional material available.

**Habitat.** There is no information available in the literature on the ecology of *Haliplusruficeps* (van Vondel and Dettner 1997). If the Lake Tonga specimen was collected, together with *H.lineatocollis*, it is likely that this species inhabits vegetated environments with slowly flowing water.

### ﻿Helophorus (Rhopalohelophorus) cf. paraminutus Angus, 1986 (Helophoridae)

A single male specimen from Lake Tonga, originally labelled *Helophorusflavipes* Fabricius, 1790, has been re-examined and reassigned. The updated determination aligns with Angus and Aouad (2009), who state that large *Helophorus* of the *minutus* group closely related to *H.paraminutus* occur in North Africa, not yet identified at species level. Furthermore, according with Angus and Aouad (2009), the record of *Helophoruslapponicus* Thomson, 1854 from Algeria (İncekara et al. 2007) should likely be attributed to these beetles. It is highly possible that this record and the male specimen from Lake Tonga here studied belongs to the same species, as the aedeagus of the latter is identical to the one illustrated in İncekara et al. (2007). However, checking the identification of this latter work is not entirely reliable without examining the original specimens, but the diagnosis of *H.lapponicus* is questionable, particularly for the size of the aedeagus illustrated (R. Angus, pers. comm. March 2025) as well as the type of environment and the altitude, at that latitude. Pending further taxonomic studies, we provisionally designate both the data of İncekara et al. (2007) and the specimen from Lake Tonga as Helophoruscf.paraminutus Angus, 1986.

**Distribution.** In its strict definition, *H.paraminutus* is known for Austria, Belarus, Czechia, European Russia, Germany, Greece, Hungary, Poland, Russia (European Russia, Western Siberia), Slovakia, Türkiye, and the United Kingdom (Przewózny 2022). The data for Tunisia (Touaylia et al. 2011a, b; Przewózny 2022) is likely referable to the H.cf.paraminutus here discussed. This represents the first record of this species complex in Algeria.

**Habitat.** Not much can be said for the specimen from Lake Tonga, presumably it was collected in still water. No description of the biotopes is given in İncekara et al. (2007) except an altitude ca 400 m a.s.l. Angus (1986) describes *H.paraminutus* as a steppe species, occurring in large numbers in shallow grassy pools left by the melting snows in spring, a very improbable habitat in North African lowlands along the coast.

### ﻿*Hydrochusgrandicollis* Kiesenwetter, 1679 (Hydrochidae)

One male and one female from Lake Tonga, in the same vial, together with the preceding species. The first record of *Hydrochusgrandicollis* for Algeria was misidentified as *H.nitidicollis* Mulsant, 1844 in İncekara and Bouzid (2007b), in which the aedeagus portrayed is indeed that of *H.grandicollis*. *Hydrochusgrandicollis* is reported also in the checklist of Lamine et al. (2022). However, Algeria is not included in the distribution of this species in the last update of the Catalogue of Palearctic Hydrophiloidea, despite being cited for Morocco and Tunisia (Przewózny 2022; Benamar et al. 2024). This find from Lake Tonga definitely confirm the presence of this species in Algeria.

**Distribution.** Southern Europe (France, Italy, Slovenia, Spain), North Africa (Algeria, Morocco, Tunisia) and Canary Islands.

**Habitat.** Still or slow-running waters. Quiet banks of streams and rivers, residual pools on riverbed.

### ﻿*Amphiopssenegalensis* (Laporte, 1840) (Hydrophilidae)

The specimens collected at Lake Tonga, labelled “*Anacaenaglobulus* (Paykull, 1798)”, represent the first record of the genus *Amphiops* in Algeria. Habitus and aedeagus of these specimens are illustrated in Figs 4, 5.

**Distribution.** A widespread species in tropical Africa, recorded also for Egypt and Morocco (Hebauer 1998; Benamar et al. 2021c; Przewózny 2022). First record for Algeria.

**Habitat.** Still waters: ponds, marshes.

### ﻿Enochrus (Methydrus) natalensis (Gemminger & Harold, 1868) (Hydrophilidae)

İncekara (2008) reported the first record of *Enochrusaffinis* (Thunberg, 1794) for northern Algeria, giving a short diagnosis of the species but without providing figures. This diagnosis closely matches the specimens of *E.natalensis* identified in our study, labelled “*Cymbiodytamarginella* (Fabricius, 1792)”. It is therefore likely that the data from İncekara (2008) actually refer to *E.natalensis* rather than *E.affinis*, although the latter species has been recorded for Morocco and Tunisia (Przewózny 2022). Habitus and aedeagus of the specimens from Lake Tonga are illustrated in Figs 6, 7. *Enochrusnatalensis* was redescribed and illustrated by Hebauer (2002), who compared it with the other Afrotropical species of the same species-group (excluding *E.affinis*, which is exclusively Palearctic). *Enochrusaffinis* and *E.natalensis* share similar size and thin apex of the parameres (but not sharply pointed in *natalensis*), bent outwards. However, *E.affinis* differs by possessing a wholly black head, without large preocular spots, and a totally black last segment of the maxillary palps. *Enochrusnigritus* (Sharp, 1873) is a third Palearctic species belonging to the subgenus Methydrus, externally very similar to both *natalensis* and *affinis*, also known from Morocco and Tunisia (Przewózny 2022). The examination of the aedeagus allows for clear differentiation of *E.nigritus* from the other two species (see Foster 1984 and Queney and Prévost 2021 for a diagnosis of *E.affinis* and *E.nigritus*). İncekaras’ Algerian record of *E.affinis* is not cited in Przewózny (2022).

**Distribution.***Enochrusnatalensis* has a subcosmopolitan distribution, occurring in the Afrotropical, Oriental, and Australian regions. In the Palearctic it is recorded from Spain, Portugal, Italy, Syria, Egypt, and Morocco (Przewózny 2022). First record for Algeria.

**Habitat.** Still waters with vegetation and debris: ponds, marshes, puddles, ditches.

### ﻿*Crephelochares ?livornicus* (Kuwert, 1890) (Hydrophilidae)

Two specimens from Lake Tonga, belonging to the genus *Crephelochares* (Fig. 8), were found stored together with specimens of *Helochareslividus* (Forster, 1771) in the same vial, labelled “*Enochrushalophilus* (Bedel, 1878)”. Unfortunately, both specimens are females, therefore a definitive species-level identification is not possible. *Crephelochareslivornicus* is the only species of the genus occurring in the Mediterranean, however, recently reported also for Tunisia (Girón and Short 2021). Compared with Italian specimens from Tuscany (type locality of *C.livornicus*), the two females from Lake Tonga exhibit a less impressed elytral punctation, consisting of finer and shallower elements. Further collection of specimens, including males, would be necessary for a definitive identification of the species.

**Distribution.***Crephelochareslivornicus* is a rare Mediterranean species, known for Bosnia and Hercegovina, Croatia, France (Corsica), Greece, Israel, Italy, Serbia and Montenegro, Spain, Tunisia, and Türkiye (Girón and Short 2021; Queney and Prévost 2021; Przewózny 2022). Although the identification of the species cannot yet be ascertained, this is the first record of the genus *Crephelochares* (as defined in Girón and Short 2021) for Algeria.

**Habitat.** Still freshwaters (ponds), mostly near the coast (Queney and Prévost 2021).

### ﻿Coelostoma (Coelostoma) hispanicum (Küster, 1848) (Hydrophilidae)

A single specimen from Lake Tonga, originally labelled “*Anacaenaglobulus* (Paykull, 1798)”, confirms the presence of *Coelostomahispanicum* in Algeria. This species was first recorded in the country by İncekara and Bouzid (2007a), despite Lamine et al. (2022) erroneously claiming to report it for the first time. Algeria is not included in the distribution of this species in the last update of the Catalogue of Palearctic Hydrophiloidea, (Przewózny 2022).

**Distribution.** Mediterranean and southern Palearctic Atlantic: Albania, Algeria, Canary Islands, Cyprus, France, Greece, Italy, Morocco, Portugal, Spain, Tunisia.

**Habitat.** Mainly banks of slow-running rivers and streams, rarely in marshy areas.

### ﻿Ochthebius (Ochthebius) fallaciosus Ganglbauer, 1901 (Hydraenidae)

Three specimens, two males and a female, originally labelled “*Hydrochuselongatus* (Schaller, 1783)”. This is the first record of *O.fallaciosus* in Algeria and confirms its presence in North Africa east of Morocco, as previously hypothesized by Jäch and Delgado (2008). The aedeagus of these specimens (Fig. 9) has the distal lobe rather wide, similar to the one illustrated from Moroccan populations in Jäch and Delgado (2008: fig. 13). *Ochthebiusviridescens* Ieniştea, 1988 is the second species belonging to the same species complex, currently known for Algeria (Jäch and Delgado 2008; Jäch and Skale 2015). *Ochthebiusfallaciosus* and *O.viridescens* are very similar externally, but easily separable for their aedeagal features (Jäch and Delgado 2008).

**Distribution.***Ochthebiusfallaciosus* has recently been given species status instead of a subspecies of *O.viridis* Peyron, 1858 (Villastrigo et al. 2018). Its distribution includes Ireland and western Great Britain, south and western France (incl. Corsica), Spain, Italy (incl. Sardinia and Sicily), Adriatic part of Croatia, Greece (Corfu); in North Africa previously known for Morocco (Jäch and Delgado 2008; Jäch and Skale 2015; Benamar et al. 2022b). First record for Algeria.

**Habitat.** Apparently seasonal ponds or marshes, both fresh and brackish.

## ﻿Discussion

A total of 42 species of aquatic Coleoptera was identified in the material collected at Lake Tonga and Garaat Djamel, belonging to ten families (Table 1). This represents approximately 13% of all species currently known from Algeria (see checklist in Suppl. material 1), with the vast majority of this diversity concentrated in Lake Tonga, underscoring its status as a key hotspot of aquatic beetle biodiversity in the region.

As expected, the water beetle fauna of both biotopes is dominated by Mediterranean elements (Afrotropico-Mediterranean, Central Asiatic-Euro-Mediterranean, Euro-Mediterranean, Mediterranean, Mediterraneo-Macaronesian, Turano-Euro-Mediterranean, West Mediterranean), which together constitute more than 50% of the chorological spectrum of the investigated localities (Table 1). A significant proportion of species also belongs to broader Holarctic and Palaearctic elements (Palearctic and West Palearctic), while Afrotropical and strictly North African species are relatively scarce.

Ecologically, species associated with still or weakly circulating waters dominate the assemblage. However, the presence of some taxa typically linked to flowing water environments and brackish waters, especially in samples from Lake Tonga indicates a mosaic of microhabitats within this complex biotope. This habitat heterogeneity likely contributes to the high species richness observed and highlights Lake Tonga as a particularly biodiverse site of ecological importance in northeastern Algeria.

The findings from Lake Tonga contribute significantly to the knowledge of the aquatic beetles in Algeria, with the addition of five species (1 Helophoridae, 3 Hydrophilidae, 1 Hydraenidae) and two genera (*Amphiops* and *Crephelochares*) new for the country. However, the identification of two of these species remains uncertain, due the still obscure taxonomic position of one and the unavailability of males of the other. Whilst it is clear that they represent two new taxa for Algeria, further studies based on additional material would be needed for a definitive diagnosis. Two species (*Hydrochusgrandicollis* and *Coelostomahispanicum*), recently recorded for Algeria (İncekara and Bouzid 2007a, b; Lamine et al. 2022) but then no longer mentioned for this country (Przewózny 2022), are here confirmed thanks to this material from Lake Tonga. Finally, the discovery of a male *Haliplusruficeps* from the same locality, marks the first documented record of this species in more than a century.

**Figures 10–13.**
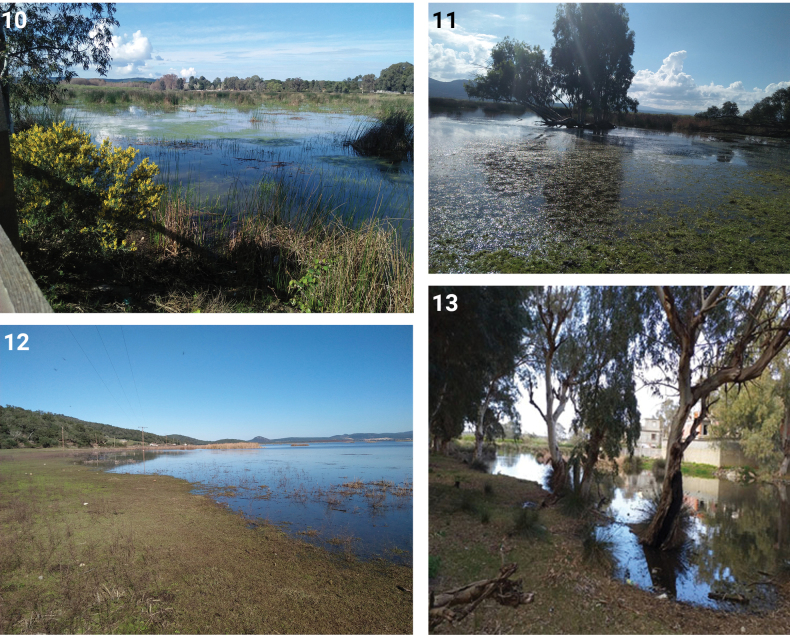
The biotopes of Lake Tonga and Garaat Djamel. **10.** Sampling Station S1 in Lake Tonga; **11.** Sampling Station S2 in Lake Tonga; **12.** Sampling Station S3 in Lake Tonga; **13.** Garaat Djamel.

Both Lake Tonga (Figs 10–12) and Gaarat Djamel (Fig. 13) are coastal lakes in the Province of El Tarf, north-eastern Algeria, not very far from each other. While the latter is a small, poorly preserved habitat, the former is one of the most ecologically intact and biodiversity-rich coastal wetlands in northern Africa, included in the El Kala National Park and designated as Ramsar site in 1983 (Mahmoudi et al. 2023, 2024). Given its ecological significance, further comprehensive surveys and taxonomic investigations are strongly encouraged to better document the full diversity of aquatic Coleoptera in this biotope. The list of species presented here likely represents only a fraction of the aquatic beetle fauna that such a vast and complex ecosystem can host. With these new findings, now Algeria counts 302 species of water beetles, sorted in 84 genera and 17 families, excluding aquatic or semiaquatic members of Chrysomelidae, Curculionidae and of other non-primary aquatic families. However, in addition to this number of taxa confirmed for the country, some recent records are still doubtful or require further verification.

A complete and up-to-date checklist of Algerian aquatic Coleoptera is provided in Suppl. material 1, with a discussion of these recent records in Suppl. material 2.

## ﻿Additional information

### Use of AI

No use of AI was reported.

### Author ORCIDs

Khaoula Mahmoudi https://orcid.org/0000-0003-4648-9668

Mario E. Toledo https://orcid.org/0000-0001-9295-3711

Fatiha Bendali-Saoudi https://orcid.org/0000-0002-2780-5330

Ilaria Negri https://orcid.org/0000-0001-5188-1408

### Data availability

All of the data that support the findings of this study are available in the main text or Supplementary Information.

## Supplementary materials

Supplementary material 1 Updated checklist of the species of water beetles known from Algeria Data type docx Explanation note This checklist is based on the revised and updated versions of the Catalogue of Palearctic Coleoptera (Löbl and Löbl 2015, 2016, 2017) and subsequent online updates. It is integrated with data from the material examined in this study as well as relevant findings from other recent publications (Bouzid and İncekara 2005; İncekara and Bouzid 2007a; 2007b; İncekara et al. 2007; İncekara 2008; Lamine et al. 2019, 2022; etc.) that were either overlooked, omitted, or published after the last updates of the Catalogue. Species not listed for Algeria in the latest updates of the Catalogue are marked with an asterisk (*), and these recently recorded for Algeria, whose presence is doubtful or requires confirmation, are enclosed in square brackets []. Exclusively North African species are marked with a (*N*) in brackets, these endemic of Algeria with an (*E*). The systematic arrangement of Coleoptera at higher classification levels follows Cai et al. 2022. https://binary.pensoft.net/file/1389928This dataset is made available under the Open Database License (http://opendatacommons.org/licenses/odbl/1.0/). The Open Database License (ODbL) is a license agreement intended to allow users to freely share, modify, and use this Dataset while maintaining this same freedom for others, provided that the original source and author(s) are credited.Khaoula Mahmoudi, Mario E. Toledo, Fatiha Bendali-Saoudi, Ilaria Negri

Supplementary material 2 Recent records of water beetles from Algeria doubtful or to be confirmed Data type doc Explanation note We provide a list of 20 recent records in Algeria that appear doubtful or that require confirmation. https://binary.pensoft.net/file/1389929This dataset is made available under the Open Database License (http://opendatacommons.org/licenses/odbl/1.0/). The Open Database License (ODbL) is a license agreement intended to allow users to freely share, modify, and use this Dataset while maintaining this same freedom for others, provided that the original source and author(s) are credited.Khaoula Mahmoudi, Mario E. Toledo, Fatiha Bendali-Saoudi, Ilaria Negri
