# Supplementary material for: ﻿Water beetles of northeastern Algeria: new records for the country and faunistic updates (Coleoptera, aquatic Adephaga, Dryopidae, Hydrophiloidea, Hydraenidae)
Source: Zookeys. 2025 Aug 7;1248:225–43. doi: 10.3897/zookeys.1248.153053 (PMC12355190; doi:10.3897/zookeys.1248.153053)
Supplement: Supplementary material 1 — Updated checklist of the species of water beetles known from Algeria [file zookeys-1248-225_article-153053__-s001.docx]

**Supplementary materials 1**

**Updated checklist of the species of water beetles known from Algeria**

This checklist is based on the revised and updated versions of the Catalogue of Palearctic Coleoptera (Löbl and Löbl 2015, 2016, 2017) and subsequent online updates. It is integrated with data from the material examined in this study as well as relevant findings from other recent publications (Bouzid and İncekara 2005; İncekara and Bouzid 2007a; 2007b; İncekara et al. 2007; İncekara 2008; Lamine et al. 2019, 2022; etc.) that were either overlooked, omitted, or published after the last updates of the Catalogue. Species not listed for Algeria in the latest updates of the Catalogue are marked with an asterisk (*), and these recently recorded for Algeria, whose presence is doubtful or requires confirmation, are enclosed in square brackets [ ]. Exclusively North African species are marked with a ***(N)*** in brackets, these endemic of Algeria with an ***(E)***. The systematic arrangement of Coleoptera at higher classification levels follows Cai et al. 2022.

Suborder **Adephaga** Clairville, 1806

Family **GYRINIDAE** Latreille, 1810 [8 spp.]

Subfamily Gyrininae Latreille, 1810

Tribe Gyrinini Latreille, 1810

Genus ***Aulonogyrus* Motschulsky, 1853**

**Subgenus** *Aulonogyrus* Motschulsky, 1853

*- concinnus* Klug, 1834

*- striatus* Fabricius, 1792

Genus ***Gyrinus* Geoffroy, 1762**

**Subgenus** *Gyrinus* Geoffroy, 1762

*- caspius* Ménétriés, 1832

*- dejeani* Brullé, 1832

*- regimbarti* Peyerimhoff, 1831

*- substriatus* Stephens, 1828

*- urinator* Illiger, 1807

Tribe Orectochilini Régimbart, 1882

Genus ***Orectochilus* Dejean, 1833**

*- villosus bellieri* Reiche, 1861

Family **Haliplidae** Aubé, 1836 [9 spp.]

Genus ***Haliplus* Latreille, 1802**

**Subgenus** *Liaphlus* Guignot, 1928

*- andalusicus* Wehncke, 1872

*- fulvus* Fabricius, 1801

*- guttatus* Aubé, 1836

*- mucronatus* Stephens, 1828

*- variegatus* Sturm, 1834

**Subgenus** *Neohaliplus* Netolitzky, 1911

*- lineatocollis* (Marsham, 1802)

*- ruficeps* Chevrolat, 1861 ***(N?)***

Genus ***Peltodytes* Régimbart, 1879**

*- caesus* (Duftschmid, 1805)

*- rotundatus* (Aubé, 1836)

Family **Noteridae** C.G. Thomson, 1860 [2 spp.]

Subfamily Noterinae Latreille, 1810

Tribe Noterini Thomson, 1860

Genus ***Canthydrus* Sharp , 1882**

*- siculus* (Ragusa, 1882)

Genus ***Noterus* Clairville, 1806**

*- laevis* Sturm, 1834

Family **Hygrobiidae** Régimbart, 1878 [1 sp.]

Genus ***Hygrobia* Latreille, 1804**

*- hermannii* (Fabricius, 1775)

Family **Dytiscidae** Leach, 1815 [98 spp. + 2 doubtful]

Subfamily Agabinae Thomson, 1867

Tribe Agabini Thomson, 1867

Genus ***Agabus* Leach, 1817**

**Subgenus** *Gaurodytes* Thomson, 1859

*- biguttatus* (Olivier, 1795)

*- bipustulatus* (Linnaeus, 1767)

*- brunneus* (Fabricius, 1798)

*- conspersus* (Marsham, 1802)

*- didymus* (Olivier, 1795)

*- dilatatus* (Brullé, 1832)

*- nebulosus* (Forster, 1771)

Genus ***Ilybius* Erichson, 1832**

*- bedeli* (Zaitzev, 1908) ***(N)***

*- chalconatus* (Panzer, 1796)

*- montanus* (Stephens, 1828)

Subfamily Colymbetinae Erichson, 1837

Tribe Colymbetini Erichson, 1837

Genus ***Colymbetes* Clairville, 1806**

*- fuscus* (Linnaeus, 1758)

*- schildknechti* Dettner, 1983

Genus ***Meladema* Laporte, 1835**

*- coriacea* Laporte, 1835

Genus ***Rhantus* Dejean, 1833**

*- hispanicus* Sharp, 1882

Subfamily Copelatinae Branden, 1884

Genus ***Liopterus* Dejean, 1833**

*- atriceps* Sharp, 1882

- *haemorrhoidalis* (Fabricius, 1878)

Subfamily Cybistrinae Sharp, 1880

Tribe Cybistrini Sharp, 1880

Genus ***Cybister* Curtis, 1827**

**Subgenus** *Cybister* Curtis, 1827

- *lateralimarginalis lateralimarginalis* (De Geer, 1774)

- *senegalensis* Aubé, 1838

- *tripunctatus africanus* Laporte, 1838

**Subgenus** *Melanectes* Brink, 1945

- *bimaculatus* Aubé, 1838

- *vulneratus* Klug, 1834

Subfamily Dytiscinae Leach, 1815

Tribe Aciliini Thomson, 1867

Genus ***Acilius* Leach, 1817**

**Subgenus** *Acilius* Leach, 1817

- *sulcatus* (Linnaeus, 1758)

Tribe Dytiscini Leach, 1815

Genus ***Dytiscus* Linnaeus, 1758**

- *circumflexus* Fabricius, 1801

- *pisanus* Laporte, 1835

- *semisulcatus* O.F. Müller, 1776

Tribe Eretini Crotch, 1873

Genus ***Eretes* Laporte, 1833**

**-** *griseus* (Fabricius, 1781)

- *sticticus* (Linnaeus, 1767)

Tribe Hidaticini Sharp, 1880

Genus ***Hydaticus* Leach, 1817**

**Subgenus** *Prodaticus* Sharp, 1882

- *bivittatus* Laporte, 1835

- *leander* (Rossi, 1790)

Subfamily Hydroporinae Aubé, 1836

Tribe Bidessini Sharp, 1880

Genus ***Bidessus* Sharp, 1880**

- *coxalis* Sharp, 1882

- *goudoti* (Laporte, 1835)

- *minutissimus* (Germar, 1823)

- *pumilus* (Aubé, 1838)

Genus ***Hydroglyphus* Motschulsky, 1853**

- *angularis* (Klug, 1834)

- *geminius* (Fabricius, 1792)

- *major* (Sharp, 1882)

- *signatellus* (Klug, 1834)

Genus ***Yola* Gozis, 1886**

- *alluaudi* Peschet, 1926

- *bicarinata bicarinata* (Latreille, 1804)

- *nigrosignata* Régimbart, 1895

Tribe Hydroporini Aubé, 1836

Subtribe Deronectina Galewski, 1994

Genus ***Deronectes* Sharp, 1882**

- *fairmairei* (Leprieur, 1876)

- *perrinae* Fery & Brancucci, 1997***(N)***

- *peyerimhoffi* Régimbart, 1906 ***(E)***

Genus ***Nebrioporus* Régimbart, 1906**

- *acuminatellus* (Fairmaire, 1876) ***(E)***

- *ceresyi* (Aubé, 1838)

- *clarkii* (Wollaston, 1862)

- *kiliani* (Peyerimhoff, 1929) ***(N)***

- *schoedli* Fery, Fresneda & Millán, 1996 ***(N)***

Genus ***Scarodytes* Gozis, 1914**

- *halensis* (Fabricius, 1787)

Genus ***Stictotarsus* Zimmermann, 1919**

- *maghrebinus* Mazzoldi & Toledo, 1998 ***(N)***

- *procerus* (Aubé, 1838)

Subtribe Hydroporina Aubé, 1836

Genus ***Hydroporus* Clairville, 1806**

- *analis* Aubé, 1838

- *distinguendus* Desbrochers des Loges, 1871

- *feryi* Wewalka, 1992 ***(N)***

- *jurjurensis* Régimbart, 1895 ***(E)***

- *limbatus* Aubé, 1838

- *lucasi* Reiche in Marseul, 1866

- *marginatus* (Duftschid, 1805)

- *memnonius* Nicolai, 1822

- *obsoletus* Aubé, 1838

- *planus* (Fabricius, 1782)

- *productus* Fairmaire, 1880 ***(N)***

- *pubescens* (Gyllenhal, 1808)

- *tessellatus* (Drapiez, 1819)

- [*tristis* (Paykull, 1798)]*

Subtribe Siettitiina Smrž, 1982

Genus ***Graptodytes* Seidlitz, 1887**

- *aurasius* (Jeannel, 1907) ***(N)***

- *exsanguis* (Bedel, 1925)

- *flavipes* (Olivier, 1795)

- *fractus* (Sharp, 1882)

- *ignotus* (Mulsant & Rey, 1861)

- *laeticulus* (Sharp, 1882) ***(N)***

- *pietrii* Normand, 1933 ***(N)***

Genus ***Metaporus* Guignot, 1945**

- *meridionalis* (Aubé, 1838)

Genus ***Porhydrus* Guignot, 1945**

- *genei* (Aubé, 1838)

Genus ***Rhithrodytes* Bameul, 1989**

- *dorsoplagiatus* (Fairmaire, 1880) ***(E)***

- *numidicus* (Bedel, 1889) ***(N)***

- [*sexguttatus* (Aubé, 1838)]*

Genus ***Stictonectes* Brink, 1943**

- *azruensis* (Théry, 1933) ***(N)***

- *escheri* (Aubé, 1838)

- *formosus* (Aubé, 1838)

- *optatus* (Seidlitz, 1887)

- *samai* Schizzerotto, 1988 ***(N)***

Genus ***Tassilodytes* Fery & Bouzid, 2016**

- *parisii* (Gridelli, 1939) ***(E)***

Tribe Hydrovatini Sharp, 1880

Genus ***Hydrovatus* Motschulsky, 1853**

- *clypealis* Sharp, 1876

- *cuspidatus* (Kunze, 1818)

Tribe Hygrotini Portevin, 1929

Genus ***Hygrotus* Stephens, 1828**

**Subgenus** *Coelambus* Thomson, 1860

- *confluens* (Fabricius, 1787)

- *pallidulus* (Aubé, 1850)

**Subgenus** *Hygrotus* Stephens, 1828

- *guineensis* (Aubé, 1838)

- *inaequalis* (Fabricius, 1777)

- *musicus* (Klug, 1834)

**Subgenus** *Leptolambus* Villastrigo, Ribera, Manuel, Millán & Fery, 2017

- *lagari* (Fery, 1992)

Tribe Hyphydrini Gistel, 1848

Genus ***Hyphydrus* Illiger, 1802**

- *aubei* Ganglbauer, 1891

- *maculatus* Babington, 1842

Tribe Methlini Branden, 1884

Genus ***Methles* Sharp, 1882**

- *cribratellus* (Fairmaire, 1880)

Subfamily Laccophilinae Gistel, 1848

Tribe Laccophilini Gistel, 1848

Genus ***Laccophilus* Leach, 1815**

- *hyalinus* (De Geer, 1774)

- *mateui* Omer-Cooper1970 ***(E)***

- *minutus* (Linnaeus, 1758)

- *poecilus* Klug, 1834

- *umbrinus* Motschulsky, 1855

Genus ***Neptosternus* Sharp, 1882**

- *ornatus* Sharp, 1882

Suborder **Myxophaga** Crowson, 1955

Family **Hydroscaphidae** LeConte, 1874 [1 sp. + 1 doubtful]

Genus ***Hydroscapha*** **LeConte, 1874**

*-* [*granulum* (Motschulsky, 1855)]*

*- mauretanica* Peyerimhoff, 1922 ***(E)***

Family **Sphaerusidae** Erichson, 1845 [1 sp.]

Genus ***Sphaerius*** **Waltl, 1838**

*- hispanicus* Matthews, 1899

Suborder **Polyphaga** Emery, 1886

Superfamily scirtoidea Fleming, 1821

Family **Scirtidae** Fleming, 1821 [15 spp.]

Subfamily Scirtinae Fleming, 1821

Genus ***Contacyphon*** **Gozis, 1886**

*- australis* (Klausnitzer, 1991) ***(N)***

*- derelictum* (Peyerimhoff, 1931)

*- lindbergi* (Nyholm, 1948)

*- ochraceus nyholmi* (Klausnitzer, 1991) ***(N)***

*- padi* (Linnaeus, 1758)

*- siculus* (Tournier, 1868)

Genus ***Elodes*** **Latreille, 1797**

*- abeillei* (Klausnitzer, 1990)

*- algirinus* (Pic, 1898) ***(E)***

*- chobauti* (Abeille de Perrin, 1990) ***(E)***

*- dubius* (Klausnitzer, 1972) ***(N)***

Genus ***Hydrocyphon*** **L.** **Redtenbacher, 1858**

*- hydrocyphonoides* (Tournier, 1868)

*- illiesi* Klausnitzer, 1991 ***(E)***

*- pallidicollis* Raffray, 1873

*- rectangulus* Klausnitzer, 1991 ***(E)***

Genus ***Prionocyphon*** **L.** **Redtenbacher, 1858**

*- numidicus* Nyholm, 1971

Superfamily dryopoidea Billberg, 1820

Family **Elmidae** Curtis, 1830 [18 spp.]

Subfamily Elminae Curtis, 1830

Tribe Elmini Curtis, 1830

Subtribe Elmina Curtis, 1830

Genus ***Elmis*** **Latreille, 1802**

*- maugetii velutina* Reiche, 1879 ***(E)***

Genus ***Esolus*** **Mulsant & Rey, 1872**

*- filum* Farmaire, 1871 ***(N)***

- *pygmaeus* P.W.J. Müller, 1806

Genus ***Limnius*** **Illiger, 1802**

- *intermedius* Fairmaire, 1881

- *opacus opacus* P.W.J. Müller, 1806

- *perrisi perrisi* (Dufour, 1843)

- *surcoufi* Pic, 1905 ***(E)***

Genus ***Oulimnius*** **Gozis, 1886**

- *fuscipes* (Reiche, 1879)

- *hipponensis* Berthélemy, 1979 ***(N)***

- *maurus* Berthélemy, 1979 ***(E)***

- *reygassei* (Peyerimhoff, 1929) ***(E)***

- *rivularis* (Rosenhauer, 1856)

- *villosus* Berthélemy, 1979 ***(N)***

Genus ***Riolus*** **Mulsant & Rey, 1872**

- *nitens* (P.W.J. Müller, 1817)

- *robustior* Pic, 1900 ***(N)***

- *substriatus* Grouvelle, 1889 ***(N)***

- *villosocostatus* (Reiche, 1879) ***(N)***

Subtribe Stenelmina Mulsant & Rey, 1872

Genus ***Stenelmis*** **Dufour, 1835**

*- consobrina consobrina* Dufour, 1835

*- leblanci* Peyerimhoff, 1929 ***(E)***

Family **Dryopidae** Billberg, 1820 [10 spp. + 3 doubtful or to be confirmed]

Genus ***Ahaggaria*** **Bollow, 1938**

*- foleyi* Peyerimhoff, 1929 ***(E)***

Genus ***Dryops*** **A.G. Olivier, 1791**

*- algiricus* (Lucas, 1846)

- *doderoi* Bollow, 1936

- *gracilis* (Karsch, 1881)

- *luridus* (Erichson, 1847)

- [*lutulentus* (Erichson, 1847)]*

*-* [*nitidulus* (Heer, 1841)]*

- *peyerimhoffi* Bollow, 1939 ***(N)***

- *seurati* Bollow, 1939 ***(E)***

- *striatellus* (Fairmaire & Brisout de Barneville, 1859)

- [*subincanus* (Kuwert, 1890)]*

- *sulcipennis* (A. Costa, 1883)

Genus ***Pomatinus*** **Sturm, 1853**

- *substriatus* (P.W.J. Müller, 1806)

Family **Limnichidae** Erichson, 1846 [2 spp.]

Subfamily Limnichinae Erichson, 1846

Genus ***Limnichus*** **Dejean, 1821**

- *aurosericeus* Jacquelin du Val, 1857

- *sericeus* (Duftschmid, 1825)

Family **Heteroceridae** W.S. Macleay, 1825 [14 spp.]

Subfamily Heterocerinae W.S. Macleay, 1825

Tribe Augylini Pacheco, 1964

Genus ***Augyles*** **Schiödte, 1866**

**subgenus** *Augyles* Schiödte, 1866

- *flavidus* (P. Rossi, 1794)

- *maritimus* (Guérin-Méneville, 1844)

- *marmota* (Kiesenwetter, 1850)

- *niloticus* (Grouvelle, 1896)

- *senescens* (Kiesenwetter, 1865)

*- turanicus* (Reitter, 1887)

Tribe Heterocerini W.S. Macleay, 1825

Genus ***Heterocerus*** **Fabricius, 1792**

**-** *aragonicus* Kiesenwetter, 1850

*- dayremi* Peyerimhoff, 1921

*- fenestratus* (Thunberg, 1784)

- *flexuosus* Stephens, 1828

- *holosericeus* Rosenhauer, 1856

- *humilis* Guillebeau, 1896

- *marginatus* (Fabricius, 1787)

- *pallidivestis* Guillebeau, 1893

Superfamily Hydrophiloidea Latreille, 1802

Family **Helophoridae** Leach, 1815 [12 + 3 doubtful or to be confirmed]

Genus ***Helophorus*** **Fabricius, 1775**

**Subgenus** *Empleurus* Hope, 1838

*- peyerimhoffi* d’Orchymont, 1926

*- porculus* Bedel, 1881

*- rufipes* (Bosch, 1791)

**Subgenus** *Eutrichelophorus* Sharp, 1915

*- oxygonus* Bedel, 1881

**Subgenus** *Helophorus* Fabricius, 1775*

*-* [*aquaticus* (Linnaeus, 1758)]*

**Subgenus** *Rhopalohelophorus* Kuwert, 1886

*- algiricus* Motshulsky, 1860 ***(N)***

*- angustatus* Motshulsky, 1860

*- asturiensis* Kuwert, 1885

*- cincticollis* Guillebeau, 1893

*- fulgidicollis* Motshulsky, 1860

*- gratus* Angus, 1987

*-* [*lapponicus* Thomson, 1854]*

*-* [*minutus* Fabricius, 1775]

- cf*. paraminutus* Angus, 1986*

First record for Algeria.

*- pici* Guillebeau, 1893

**Subgenus** *Trichohelophorus* Kuwert, 1886

*- alternans* Gené, 1836

Family **Georissidae** Laporte, 1840 [1 sp.]

Genus ***Georissus*** **Latreille, 1809**

**Subgenus** *Neogeorissus* Satô, 1972

*- costatus* Laporte, 1840

Family **Hydrochidae** Thomson, 1859 [2 spp. + 2 doubtful or to be confirmed]

Genus ***Hydrochus*** **Leach, 1817**

*-* [*flavipennis* Küster, 1852]*

*- grandicollis* Kiesenwetter, 1870*

- [*nitidicollis* Mulsant, 1844]*

*- smaragdineus* Farmaire, 1879

Family **Hydrophilidae** Latreille, 1802 [50 spp. + 5 doubtful or to be confirmed]

Subfamily Hydrophilinae Latreile, 1802

Tribe Amphiopini Kuwert, 1890

Genus ***Amphiops*** **Erichson, 1843***

*- senegalensis* (Laporte, 1840)*

First record for Algeria.

Tribe Berosini Mulsant, 1844

Genus ***Berosus*** **Leach, 1843**

**Subgenus** *Berosus* Leach, 1843

*- affinis* Brullé, 1835

*- fuscostriatus* Farmaire, 1892

*- hispanicus* Küster, 1847

*- signaticollis* (Charpentier, 1825)

**Subgenus** *Enoplurus* Hope, 1838

*- bispina* Reiche & Saulcy, 1856

*- furcatus* Boheman, 1851

*- guttalis* Rey, 1883

Tribe Laccobiini Houlbert, 1922

Genus ***Laccobius*** **Erichson, 1837**

**Subgenus** *Dimorpholaccobius* Zaitzev, 1938

*- atrocephalus atrocephalus* Reitter, 1872

*- hispanicus* Gentili, 1974

*- moraguesi* Régimbart, 1898

*- neapolitanus* Rottenberg, 1874

*- sinuatus sinuatus* Motschulsky, 1849

*- syriacus* Guillebeau, 1896

**Subgenus** *Hydroxenus* Wollaston, 1867

*- femoralis mulsanti* Zaitzev, 1908

*- revelieri* Perris, 1864

**Subgenus** *Microlaccobius* Gentili, 1974

*- algiricus* M. Hansen, 1999

*- gracilis gracilis* Motschulsky, 1855

*- praecipuus* Kuwert, 1890

**Subgenus** *Notoberosus* Blackburn, 1895

*- pommayi* Bedel, 1881 ***(N)***

Genus ***Paracymus*** **Thomson, 1867**

*-* [*aeneus* (Germar, 1824)]*

*- relaxus* Rey, 1884

*- scutellaris* (Rosenhauer, 1856)

Tribe Hydrobiusini Mulsant, 1844

Genus ***Hydrobius*** **Leach, 1815**

*- fuscipes* (Linnaeus, 1758)

- [*articus* Kuwert, 1890]*

Genus ***Limnohydrobius*** **Reitter, 1909**

*- convexus* (Brullé, 1835)

Genus ***Limnoxenus*** **Motschulsy, 1853**

*- niger* (Gmelin, 1790)

Tribe Hydrophilini Latreille, 1802

Genus ***Hydrophilus*** **Geoffroy, 1762**

**Subgenus** *Hydrophilus* Geoffroy, 1762

*- piceus* (Linnaeus, 1758)

*- pistaceus* Laporte, 1840

*- senegalensis* Percheron, 1835

Genus ***Sternolophus*** **Solier, 1834**

*- solieri* Laporte, 1840

Subfamily Chaetarthriinae Bedel, 1881

Tribe Anacaenini M. Hansen, 1991

Genus ***Anacaena*** **Thomson, 1859**

*- bipustulata* (Marsham, 1802)

*- globulus* (Paykull, 1798)

*- limbata* (Fabricius, 1792)

*- lutescens* (Stephens, 1829)

Tribe Chaetarthrinii Bedel, 1881

Genus ***Chaetarthria*** **Stephens, 1835***

*-* [*seminulum* (Herbst, 1797)]*

Genus ***Hemisphaera*** **Pandellé, 1876***

*-* [*seriatopunctata* (Perris, 1874)]*

Subfamily Enochrinae Short & Fikáček, 2013

Genus **[*Cymbiodyta*** **Thomson, 1859]***

*-* [*marginella* (Fabricius, 1792)]*

Genus ***Enochrus*** **Bedel, 1881**

**Subgenus** *Enochrus* Thomson, 1859

*- melanocephalus* (Olivier, 1793)

**Subgenus** *Lumetus* Zaitzev, 1908

*- ater* (Kuwert, 1888)

*- bicolor* (Fabricius, 1792)

*- politus* (Küster, 1849)

*- segmentinotatus* (Kuwert, 1888)

**Subgenus** *Methydrus* Rey, 1885*

*-* [*affinis* (Thunberg, 1794)]*

*- natalensis* (Gemminger & Harold, 1868)*

First record for Algeria.

Subfamily Acidocerinae Zaitzev, 1908

Genus ***Crephelochares*** **Kuwert, 1890***

- ?*livornicus* (Kuwert, 1890)*

First record for Algeria.

Genus ***Helochares*** **Mulsant, 1844**

*- lividus* (Forster, 1771)

Subfamily Sphaeridiinae Latreille, 1802

Tribe Coelostomatini L. Heiden, 1891

Genus ***Coelostoma*** **Brullé, 1835***

**Subgenus** *Coelostoma* Brullé, 1835*

*- hispanicum* (Küster, 1848)*

Genus ***Dactylosternum*** **Wollaston, 1854**

*- abdominale* (Fabricius, 1792)

Tribe Megasternini Mulsant, 1844

Subtribe Megasternina Mulsant, 1844

Genus ***Cercyon*** **Leach, 1817**

**Subgenus** *Cercyon* Leach, 1817

*- haemorrhoidalis* (Fabricius, 1775)

*- obsoletus* (Gyllenhal, 1808)

*- terminatus* (Marsham, 1802)

**Subgenus** *Paracercyon* Seidlitz, 1888

*- analis* (Paykull, 1798)

Genus ***Megasternum*** **Mulsant, 1844**

*- concinnum* (Marsham, 1802)

Tribe Sphaeridiini Latreille, 1802

Genus ***Sphaeridium*** **Fabricius, 1775**

*- bipustulatum* Fabricius, 1781

*- lunatum* Fabricius, 1792

*- marginatum* Fabricius, 1787

Superfamily staphylinoidea Latreille, 1802

Family **hydraenidae** Mulsant, 1844 [57 spp. + 3 doubtful]

Subfamily Hydraeninae Mulsant, 1844

Tribe Hydraenini Mulsant, 1844

Genus ***Hydraena*** **Kugelann, 1794**

**Subgenus** *Hydraena* Kugelann, 1794

*- algerina* Kaddouri, 1992 ***(E)***

*- audisioi* Jäch, 1992 ***(E)***

*- bedeli* Berthélemy, 1992 ***(E)***

*- chobauti* Guillebeau, 1896 ***(E)***

*- cordata* L.W. Schaufuss, 1883

*- explanata* Pic, 1905 ***(E)***

*- kocheri* Berthélemy, 1992 ***(N)***

*- leprieuri* Sainte-Claire Deville, 1905 ***(N)***

*- mouzaiensis* Sainte-Claire Deville, 1909 ***(E)***

*- numidica* Sainte-Claire Deville, 1905 ***(N)***

*- pici* Sainte-Claire Deville, 1905 ***(N)***

*- rigua* d’Orchymont, 1931 ***(N)***

*- rivularis* Guillebeau, 1896 ***(N)***

*- scabrosa* d’Orchymont, 1931 ***(N)***

**Subgenus** *Hydraenopsis* Janssens, 1972

*- quadricollis* Wollaston, 1864

**Subgenus** *Phothydraena* Kuwert, 1888

*- atrata* Desbrochers des Loges, 1891

*- pallidula* Sainte-Claire Deville, 1909 ***(E)***

*- testacea* Curtis, 1830

Tribe Limnebiini Mulsant, 1844

Genus ***Limnebius*** **Leach, 1815**

**Subgenus** *Bilimneus* Rey, 1883

*- evanescens* Kiesenwetter, 1866

*- nanus* Jäch, 1993

**Subgenus** *Limnebius* Leach, 1815

*- nitifarus* d’Orchymont, 1938 ***(N)***

*- pilicauda* Guillebeau, 1896

*- theryi* Guillebeau, 1891 ***(N)***

Subfamily Ochthebiinae Thomson, 1859

Genus ***Ochthebius*** **Leach, 1815**

**Subgenus** *Asiobates* Thomson, 1859

*- abeillei* Guillebeau, 1896

*- aeneus* Stephens, 1835

*- bonnairei* Guillebeau, 1896

*- dilatatus* Stephens, 1829

*- immaculatus* Breit, 1908

*- maculatus* Reiche, 1872

**Subgenus** *Aulacochthebius* **Kuwert, 1887**

*- exaratus* Mulsant, 1844

**Subgenus** *Calobius* Wollaston, 1854

*- quadricollis* Mulsant, 1844

**Subgenus** *Cobalius* Rey, 1886

- *subinteger* Mulsant & Rey, 1861

**Subgenus** *Ochthebius* Leach, 1815

*- atriceps* Fairmaire, 1879 ***(N)***

*- auropallens* Fairmaire, 1879

- *bifoveolatus* Waltl, 1835*

First record for Algeria in Lamine et al. (2019).

*- cuprescens* Guillebeau, 1893

*- difficilis* Mulsant, 1844

*- fallaciosus* Ganglbauer, 1901*

First record for Algeria.

*- gauthieri* Peyerimhoff, 1924 ***(N)***

*- grandipennis* Fairmaire, 1879

*- kieneri* Jäch, 1999 ***(E)***

- [*lobicollis* Rey, 1885]*

*- mauretanicus* Jäch, 1990 ***(N)***

*- mediterraneus* (Ieniştea, 1988)

*- meridionalis* Rey, 1885

*- merinidicus* Ferro, 1985

*- normandi* Jäch, 1992 ***(N)***

- *notabilis* Rosenhauer, 1865

- [*pedicularius* Kuwert, 1887]*

*- pilosus* Waltl, 1835

*- poweri* Rye, 1869

*- praetermissus* Jäch, 1991 ***(N)***

*- punctatus* Stephens, 1829

*- quadrifossulatus* Waltl, 1835

*- quadrifoveolatus* Wollaston, 1854

- [*semisericeus* Sainte-Claire Deville, 1914]*

*- subpictus* Wollaston, 1857

*- tacapasensis tacapasensis* Ferro, 1983

*- velutinus* Fairmaire, 1883

*- viridescens* Ieniştea, 1988
