# Supplementary material for: ﻿Water beetles of northeastern Algeria: new records for the country and faunistic updates (Coleoptera, aquatic Adephaga, Dryopidae, Hydrophiloidea, Hydraenidae)
Source: Zookeys. 2025 Aug 7;1248:225–43. doi: 10.3897/zookeys.1248.153053 (PMC12355190; doi:10.3897/zookeys.1248.153053)
Supplement: Supplementary material 2 — Recent records of water beetles from Algeria doubtful or to be confirmed [file zookeys-1248-225_article-153053__-s002.doc]

**Supplementary material 2**

***Recent records of water beetles from Algeria doubtful or to be confirmed***

***Hydroporus tristis*** (Paykull, 1798) (Dytiscidae). In Lamine et al. (2022), this species is recorded as new for Algeria without providing diagnostic information or illustrations. *H. tristis* is a Holarctic species, widespread in Europe, Russia, Japan, and North America (Nilsson and Hájek 2025), but its presence in North Africa is highly improbable. In the above paper, *H. tristis* is mentioned as frequent in the studied area (northern Algeria), therefore, this record should almost certainly be referred to another species.

***Graptodytes varius*** (Aubé, 1838) (Dytiscidae). This species was known for Algeria in its varieties *laeticulus* (Sharp, 1882) and *exsanguis* (Bedel, 1925), although later Guignot (1959b) synonymized the latter with *Graptodytes fractus* (Sharp, 1882). Both *laeticulus* and *exsanguis* have recently been given valid species status and actually *G. varius* is considered a southern European species absent from North Africa, except Morocco (Fery and Bouzid 2016; Queney and Manuel 2019; Benamar et al. 2021a; Nilsson and Hájek 2025). However, in Benamar et al. (2021a) *G. varius* is still mentioned for Algeria and Tunisia, whereas Lamine et al. (2022) give an Algerian record of this species, which likely refers to *G. laeticulus*.

***Rhithrodytes sexguttatus*** (Aubé, 1838) (Dytiscidae). Mentioned for Algeria in Lamine et al. (2022), this is a species endemic of the Tyrrhenian Islands (Corsica, Sardinia, Tuscan Archipelago) and with any probability the data in Lamine et al. (2022) must be referred to *R. dorsoplagiatus* (Fairmaire, 1880), *R. minimus* Fery, 2016 or *R. numidicus* (Bedel, 1889).

***Hydroscapha granulum*** (Motschulsky, 1855) (Hydroscaphidae). Recorded as new for Algeria in Lamine et al. (2022), without diagnosis or illustrations. A southeastern European species, recorded also for Türkiye and Iran, but not for North Africa (Hájek 2017a). In the same paper it is mentioned also a second, more widespread, unidentified species (*Hydroscapha* sp.), which might be referred to *H. mauretanica* Peyerimhoff, 1922, endemic to Algeria (Hájek 2017a).

***Dryops lutulentus*** (Erichson, 1847) (Dryopidae). Mentioned for Algeria in Lamine et al. (2022), although this species has not been reported for Algeria in previous works. Europe, Central Asia, and Arabian Peninsula; in North Africa known only for Morocco (Kodada and Jäch 2016; Benamar et al. 2022a). Its presence in Algeria is probable but need to be confirmed.

***Dryops nitidulus*** (Heer, 1841) (Dryopidae). Recorded as new for Algeria in Lamine et al. (2022), without further information. A mainly central European species, not known from North Africa (Kodada and Jäch 2016), its presence in this territory is rather improbable.

***Dryops subincanus*** (Kuwert, 1890) (Dryopidae). As for *D. lutulentus,* in Lamine et al. (2022)*.* An Europeo-Mediterranean species, in North Africa known for a generic record (“North Africa”) (Kodada and Jäch 2016). However, its presence in Algeria is possible.

***Helophorus* (*Helophorus*) *aquaticus*** (Linnaeus, 1758) (Helophoridae). Recorded as new for Algeria in İncekara et al. (2007). The aedeagus figured in this publication would fall more into the range of *H. aequalis* Thomson, 1868 rather than *H. aquaticus.* In agreement with Robert Angus (personal communication), it is not possible to confirm or deny İncekaras’ identification. However, it is indeed the first record of the subgenus *Helophorus* s. str. in Algeria. In the checklist of Boukli Hacene et al. (2012) it is cited *“Helophorus* gr. *maritimus* Rey, 1885” [sic!], which might represent the second record of this subgenus from Algeria. *Helophorus aquaticus* is a Sibero-European species, known also from Morocco (Przewózny 2022).

***Helophorus* (*Rhopalohelophorus*) *lapponicus*** Thomson, 1854 (Helophoridae). Recorded as new for Algeria in İncekara et al. (2007). A Boreal species from East Siberia to northern Europe with relict, high altitude populations in southern Europe and Middle Orient (Angus 1986, 2020; Przewózny 2022). With all likelihood the identification in İncekara et al. (2007) is incorrect and the species does not occur in North Africa (see discussion here above in *Helophorus* cf. *paraminutus* from Lake Tonga).

***Helophorus* (*Rhopalohelophorus*) *minutus*** Fabricius, 1775 (Helophoridae). A Mainly European species, formally recorded for Algeria and Morocco (Przewózny 2022). Accordingly with Angus and Aouad (2009) and Shatrovskiy and Angus (2024) all data of *H. minutus* in North Africa need to be revised and perhaps the true *H. minutus* does not occur in this area.

***Hydrochus flavipennis*** Küster, 1852 (Hydrochidae). Mentioned for Algeria in İncekara and Bouzid (2007b). Nevertheless, this species has not been reported from Algeria in previous works, nor cited later. *H. flavipennis* is a Turano-European species, recorded also from Morocco and Tunisia (Przewózny 2022; Benamar et al. 2024) and its presence in Algeria is at least probable, although it is not possible to know what the author’s quote is based on.

***Hydrochus nitidicollis*** Mulsant, 1844 (Hydrochidae). The first record of this species in Algeria (Incekara ad Bouzid 2007b) is obviously based on a misidentification (see discussion in *Hydrochus grandicollis* in the text). The record of *H. nitidicollis* in Algeria is restated in Lamine et al. (2022), without further comments, and in Benamar et al. (2024). *Hydrochus nitidicollis* is a western European species, recorded from North Africa as a generic “Northwest Africa” and an old, unreliable, record from Morocco (Przewózny 2022; Benamar et al. 2024). The presence of this species in Algeria, as well as in the whole North Africa is yet to be proved.

***Paracymus aeneus*** (Germar, 1824) (Hydrophilidae). This species is mentioned in Lamine et al. (2022), although it was not reported from Algeria before. A Central Asiatic-European-Mediterranean species, in North Africa it was already recorded from Egypt, Morocco and Tunisia (Przewózny 2022), therefore its presence in Algeria is probable. *Paracymus aeneus* preferentially inhabits brackish stagnant waters near the coast, however, in Lamine et al. (2022) the species is treated as inhabiting mountain rivers with shady courses, therefore it is possible that this work refers to another species.

***Hydrobius arcticus*** Kuwert, 1890 (Hydrophilidae). Recorded in İncekara (2008) as new for Algeria. The presence of this boreal species in North Africa is highly unlikely, although not impossible, localised populations apparently referrable to this taxon being known in alpine environments in Türkiye and Iran (Hansen 1987; Fossen et al. 2016; Przewózny 2022). In İncekara (2008) a short diagnosis of the species is given, without illustrations, but the characters described do not allow a safe identification. The locality of this record is also at low altitude (ca. 400 m a.s.l.) near the coast, which makes the data even less convincing.

***Chaetarthria seminulum*** (Herbst, 1797); ***Hemisphaera seriatopunctata*** (Perris, 1874) (Hydrophilidae). Both these genera of Chaetarthrinii are cited for the first time in Algeria in Lamine et al. (2022). *Chaetarthria seminulum* is recorded as new for the country, *Hemisphaera seriatopunctata* not. However, the genus *Hemisphera* had not been recorded from Algeria previously, as well as *Chaetarthria*, although both already known from Morocco and, the latter, also Tunisia (Przewózny 2022). Besides, In Lamine et al. (2022) no diagnostic information is given, therefore it is not possible to know for sure whether the specific identifications are correct or should be referred to other species of these genera, also known in North Africa (Benamar et al. 2021c; Przewózny 2022).

***Cymbiodyta marginella*** (Fabricius, 1792) (Hydrophilidae). Recorded as new for Algeria in Lamine et al. (2022), without diagnostic information. An Algerian record of this species is published also in Mahmoudi et al. (2023), but we have here ascertained that it is a misidentification of *Enochrus natalensis* (Gemminger and Harold, 1868). *Cymbiodyta marginella* has a Sibero-European distribution (Przewózny 2022); currently, it is yet to be proved that it occurs in Algeria as well as in North Africa.

***Enochrus* (*Methydrus*) *affinis*** (Thunberg, 1794) (Hydrophilidae). Recorded in İncekara (2008) as new for Algeria. This record needs to be confirmed, and it might be referred to *E. natalensis*.

***Ochthebius* (*Ochthebius*) *lobicollis*** Rey, 1885 (Hydraenidae). Mentioned in Lamine et al. (2022), although it was not reported from Algeria before. It is a north-western Mediterranean species (Ribera and Hernando 2019) the presence of which in Algeria is improbable. *Ochthebius mauretanicus* Jäch, 1990, belongs to the same species group (sensu Jäch 1990) and is perhaps likely this Algerian record of *O. lobicollis* may refer to this species.

***Ochthebius* (*Ochthebius*) *pedicularius* Kuwert**, 1887 (Hydraenidae). Recorded as new for Algeria in Lamine et al. (2022), without diagnostic information. It is a Central-East European species (Jäch and Skale 2015) and its presence in North Africa is highly improbable. Five species belonging to the same species-group (*O. foveolatus* group, sensu Jäch 1991) are known from Algeria (*O. gauthieri* Peyerimhoff, 1924, *O. mediterraneus* Ieniştea, 1988, *O. merinidicus* Ferro, 1985, *O. praetermissus* Jäch, 1991, *O. tacapasensis* Ferro, 1983) and the record of *O. pedicularius* may refer to one of them.

***Ochthebius* (*Ochthebius*) *semisericeus*** Sainte-Claire Deville, 1914 (Hydraenidae). Mentioned in Lamine et al. (2022), although it was not reported for Algeria before. Known only for France and Spain, perhaps this species is not present in North Africa (Jäch 2001; Jäch and Skale 2015), but a number of yet unidentified species of the same species-complex would occur, some of which very closely related to *O. griotes* Ferro, 1985, a species currently considered endemic to Morocco (Jäch and Skale 2015; Mabrouki et al. 2018; Benamar et al. 2022b).
